# Supplementary material for: The Involvement of Antioxidant Enzyme System, Nitrogen Metabolism and Osmoregulatory Substances in Alleviating Salt Stress in Inbred Maize Lines and Hormone Regulation Mechanisms
Source: Plants (Basel). 2022 Jun 10;11(12):1547. doi: 10.3390/plants11121547 (PMC9227288; doi:10.3390/plants11121547)
Supplement: Supplementary file 1 [file plants-11-01547-s001.zip › plants-1738402-supplementary.pdf]

Supplementary Materials:

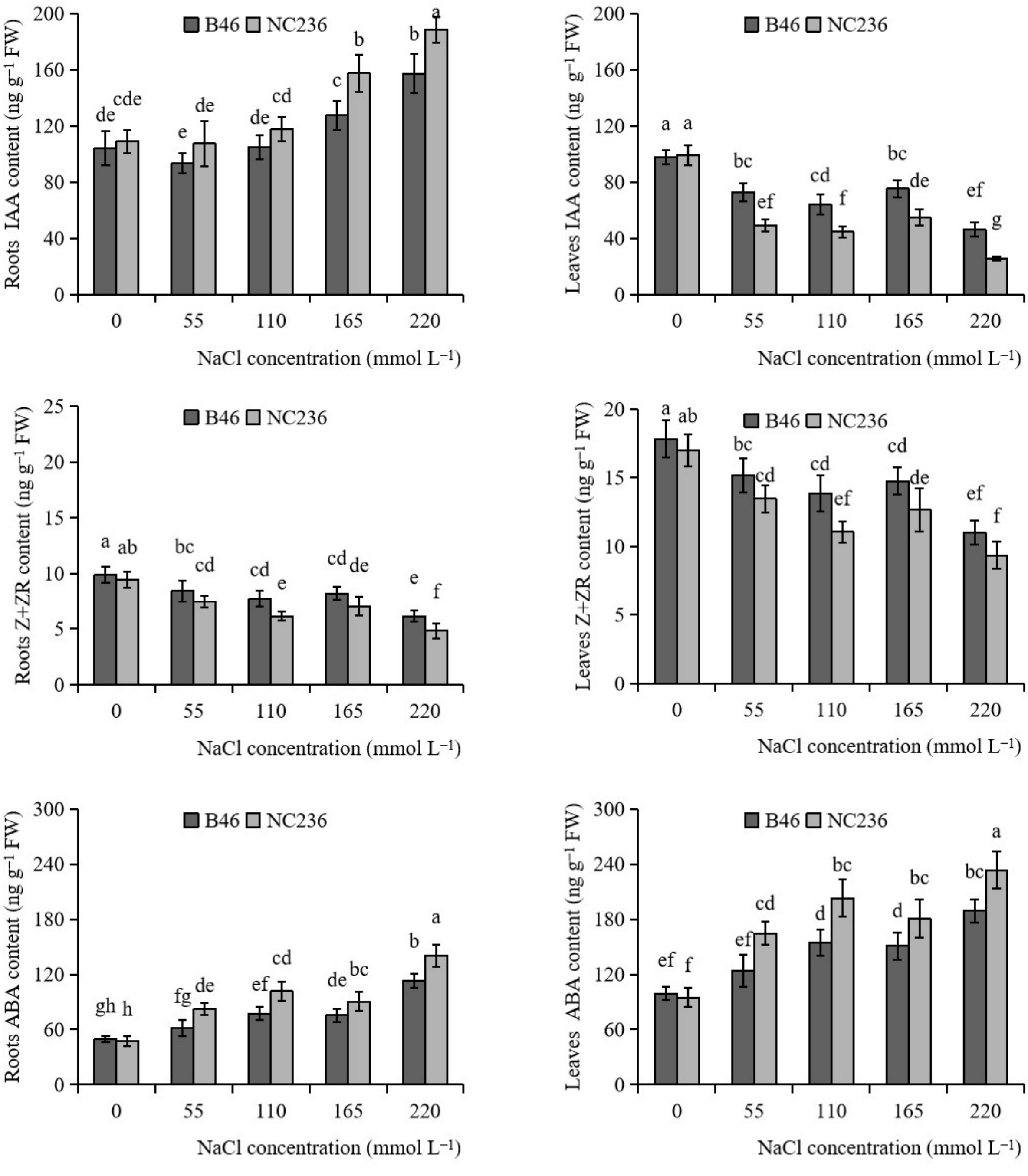

Figure S1. Effects of salt stress on the contents of IAA, Z+ZR, and ABA in maize roots and leaves. Data are expressed as mean ± standard deviation. Different letters within the same column indicate significant difference at the 5% level.

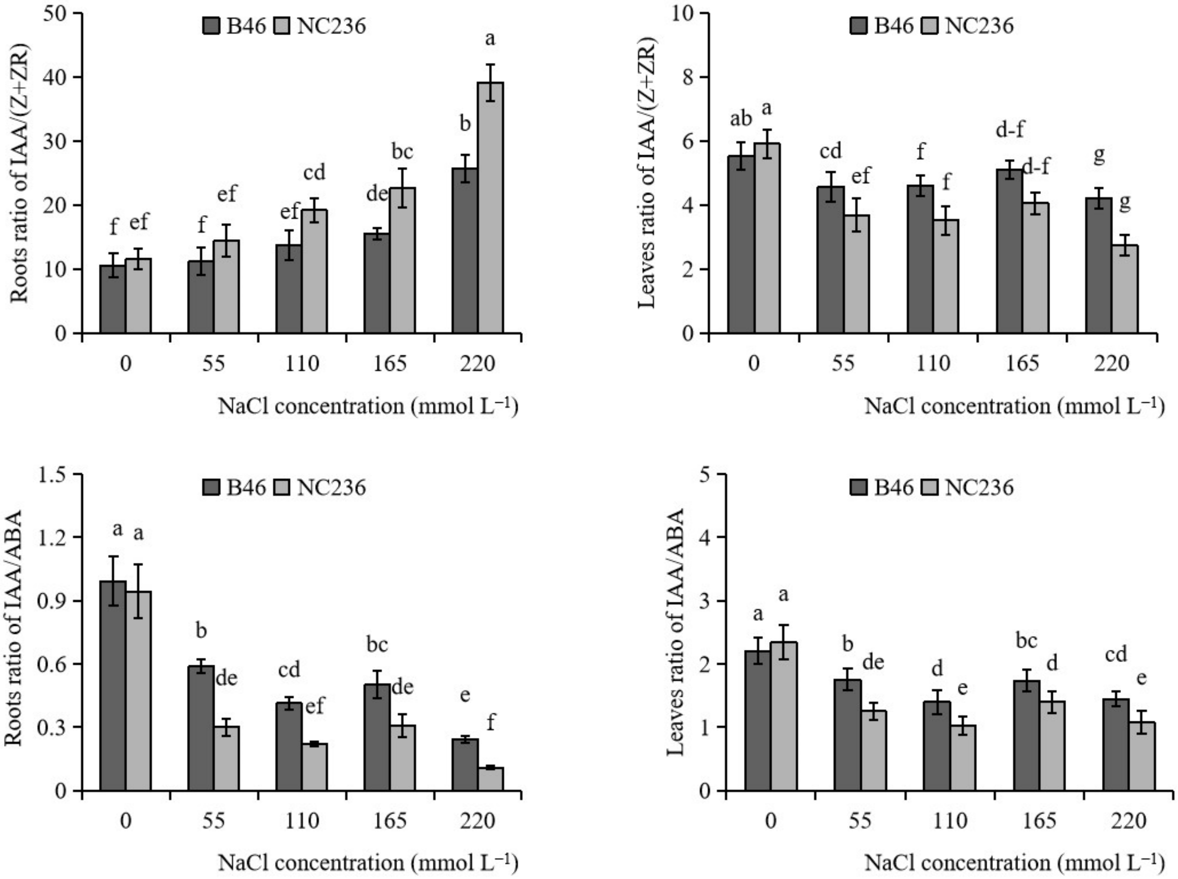

Figure S2. Effects of salt stress on the ratio of IAA/(Z+ZR) and IAA/ABA in maize roots and leaves. Data are expressed as mean ± standard deviation. Different letters within the same column indicate significant difference at the 5% level.
